# Evaluation of Precise Microwave Ranging Technology for Low Earth Orbit Formation Missions with Beidou Time-Synchronize Receiver

**DOI:** 10.3390/s21144883

**Published:** 2021-07-17

**Authors:** Xiaoliang Wang, Shufan Wu, Deren Gong, Qiang Shen, Dengfeng Wang, Christopher Damaren

**Affiliations:** 1 School of Aeronautics and Astronautics, Shanghai Jiao Tong University, Shanghai 200240, China; xlwang12321@sjtu.edu.cn (X.W.); drgong@sjtu.edu.cn (D.G.); qiangshen@sjtu.edu.cn (Q.S.); 2 Institute of Space Radio Technology, Xi’an 710100, China; dfwang_aero@163.com; 3 Institute for Aerospace Studies, University of Toronto, Toronto, ON M1C 1A4, Canada; damaren@utias.utoronto.ca

**Keywords:** spacecraft formation flying, low Earth orbit, microwave ranging, relative navigation, Earth’s gravity field, digital elevation models

## Abstract

In this study, submillimeter level accuracy K-band microwave ranging (MWR) equipment is demonstrated, aiming to verify the detection of the Earth’s gravity field (EGF) and digital elevation models (DEM), through spacecraft formation flying (SFF) in low Earth orbit (LEO). In particular, this paper introduces in detail an integrated BeiDou III B1C/B2a dual frequency receiver we designed and developed, including signal processing scheme, gain allocation, and frequency planning. The receiver matched the 0.1 ns precise synchronize time-frequency benchmark for the MWR system, verified by a static and dynamic test, compared with a time interval counter synchronization solution. Moreover, MWR equipment ranging accuracy is explored in-depth by using different ranging techniques. The test results show that MWR achieved 40 μm and 1.6 μm/s accuracy for ranging and range rate during tests, using synchronous dual one-way ranging (DOWR) microwave phase accumulation frame, and 6 μm/s range rate accuracy obtained through a one-way ranging experiment. The ranging error sources of the whole MWR system in-orbit are analyzed, while the relative orbit dynamic models, for formation scenes, and adaptive Kalman filter algorithms, for SFF relative navigation designs, are introduced. The performance of SFF relative navigation using MWR are tested in a hardware in loop (HIL) simulation system within a high precision six degree of freedom (6-DOF) moving platform. The final estimation error from adaptive relative navigation system using MWR are about 0.42 mm (range/RMS) and 0.87 μm/s (range rate/RMS), which demonstrated the promising accuracy for future applications of EGF and DEM formation missions in space.

## 1. Introduction

Spacecraft formation flying has attracted much attention since it can perform space missions with more reliability, adaptability, and low life-cycle cost, compared with traditional monolithic spacecraft [1,2]. Several SFF missions have been successfully deployed in low Earth orbit, such as GRACE and GRACE Follow-on mission for precise Earth gravity field measurement [3,4,5,6], EO-1/LandSat 7 for Earth observation [7], and PRISMA for millimeter level SFF technology in-orbit demonstration [8].

Among the many LEO SFF space applications, the most frequently used ones are the Earth’s gravity field detection through follow-on formation [9], and digital elevation models mapping by using pendulum formation configurations. EGF is one basic physical field, that reflects the influences on the distribution and movement of the Earth’s materials. The EGF and its time variation reflect the density distribution and material movement state of the earth’s surface and interior, and determine the fluctuation and variation of geoid. The high-precision EGF model can be used for space technology, in aerospace engineering with a requirement of accuracy and resolution. Currently, DEM are a key data source and play an important role in a wide range of environmental applications [10,11], such as TanDEM-X formation mission for the generation of high-precision digital elevation models, by using a high-resolution interferometric synthetic-aperture radar (In-SAR) [12,13].

For those SFF space science missions, one of the major tasks is verifying a precise guidance, navigation, and control (GNC) system in-orbit, and, most important, highly accurate inter-satellite-link (ISL) baseline ranging technology. The ranging technique with high accuracy can be realized through different approaches, such as a laser interferometric technique for the mapping of Earth’s gravity field, or gravitational wave detection [14,15,16,17]. Application of a laser interferometric technique can be found in the LISA-Pathfinder mission, launched in December 2015, lead by NASA and ESA [17,18,19], and GRACE follow-on mission, launched in May 2018 [20,21,22,23]. At the same time, a microwave radio based ranging technique is also widely used in space, and even functioned as the primary ranging payload in real observing missions [24,25].

In the past few years, we have conducted arduous work on the designing, development, and testing of microwave ranging (MWR) equipment that achieves submillimeter level ranging accuracy, ready for reconfigurable LEO SFF Earth’s gravity field detection (EGFD) mission that scheduled launch within next two years. Similar but different from GRACE, EGFD is a multi-functional LEO SFF mission that preliminary designed in different formation configurations with relative distance of tens to hundreds of kilometers, according to the real task in-orbit. Moreover, applications of MWR to DEM missions with pendulum formation configuration are also considered.

Additionally, with the original observe data obtained, the most forecast reward of EGFD mission is the design and development of submillimeter level accuracy ranging equipment with in-orbit verification, which enables the future application of a precise GNC system for SFF missions in both LEO and HEO scenario [26]. The K-band MWR payload is particularly designed and deployed for reasons below:

1. Unlike accuracy optical ranging sensors that relies on high precision pointing mechanism [22,27], microwave ranging can be realized by transceiver antenna with a few degrees wide main lobe angle. At the same time, microwave ranging can operate in a pseudo-code mode or carrier mode for coarse or precise ranging if necessary, as required by mission control, providing a flexible solution to the GNC system;
2. Good inheritance of microwave ranging technology from existing space experience [28];
3. As an essential supplement to the MWR system, BeiDou III B1C/B2a dual frequency navigation signal receiving and processing technology can be fully tested and verified in an SFF mission. The receiver can provide high accuracy time synchronization solutions, with precision to a nanosecond, served as a time scale benchmark between formation satellites. Moreover, the receiver can provide precise stand-alone navigation solutions using a precise orbit determination algorithm;
4. Microwaves can also be used for real-time data transmission between spacecrafts for original science data exchange, for differential GPS measurement data transmission for relative navigation, and, possibly, as a backup channel for mission telemetry, track, and command (TT&C) system.

The following content is organized as follows: Section 2 introduces the submillimeter level accuracy microwave ranging technique and the measurement equipment development process. The designing and development process of the BeiDou III dual frequency receiver for time synchronization is provided in Section 3. Section 4 describes the ranging performance test and adaptive relative navigation filter algorithm simulation under a real SFF mission scenario.

## 2. Earth’s Gravity Field Detection Mission and Payloads

### 2.1. EGFD Mission Analysis

EGFD is a typical LEO SFF mission that is conducted by using follow-on formation configuration in space. Three major payloads are used, which include: high sensitivity three-axis accelerometer, dual frequency BeiDou receiver, and high precision K-band inter-satellite microwave ranging equipment. The principle of retrieving the high precision EGF data from EGFD mission is to accurately measure the relative orbit perturbation of two in-orbit follow-on formation flying spacecrafts, and eliminate the influence of various non-conservative forces (such as atmospheric resistance, solar radiation pressure, satellite orbit maneuver, etc.) through the separation of measurement from on-board accelerometers, so as to deduce the distribution characteristics of the gravity field.

The basic ways to retrieve the EGF data are as follows [29]: first, MWR is used to accurately measure the line of sight (LOS) distance change and its rate between the antenna phase centers of two LEO follow-on formation satellites, and then calculate the distance change and its rate between the mass centers of two formation satellites. Second, the accelerometers, fixed on the center of mass in both formation satellites, are used to accurately measure the non-conservative forces. The MWR measurement information is accurately calculated and converted to centroids of two formation satellites, by attitude measurement, satellite platform design, and MWR antenna installation. Then, the distance change and its rate between the two satellite centroids caused by conservative force can be obtained. The dual frequency BeiDou receiver is used for MWR system time synchronization and precise orbit determination, and the distribution characteristics of the EGF can be finally deduced. The following is a detailed task analysis of these two aspects.

#### 2.1.1. Measurement of LOS Distance and Its Rate between Antenna Phase Centers by MWR

Basically, the MWR system is a synchronous dual one-way ranging (DOWR) microwave phase accumulation ranging system [30]. By transmitting and receiving microwave signals in K/Ka band between two leader-follower satellites, the offset distance between the antenna phase centers of two formation satellites is measured by using integral Doppler (integral Doppler measures the phase accumulation information based on a certain period of time, that is, it contains the distance change information based on a certain time starting point, called biased distance $\Delta \rho (t-t_{0})$, in the distinguishing of absolute distance $\rho (t_{0})$), and then the first and second order variation of the distance are obtained.

#### 2.1.2. Calculation of the LOS Distance Change and Its Rate between Centroids

**Figure 1:**
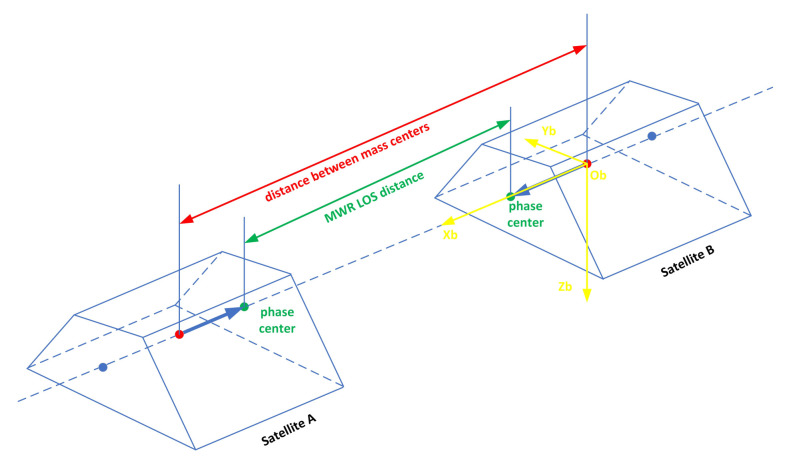
The geometry of LOS distance between leader-follower satellite antenna centers and distance between mass centers (ideal situation).

**Figure 2:**
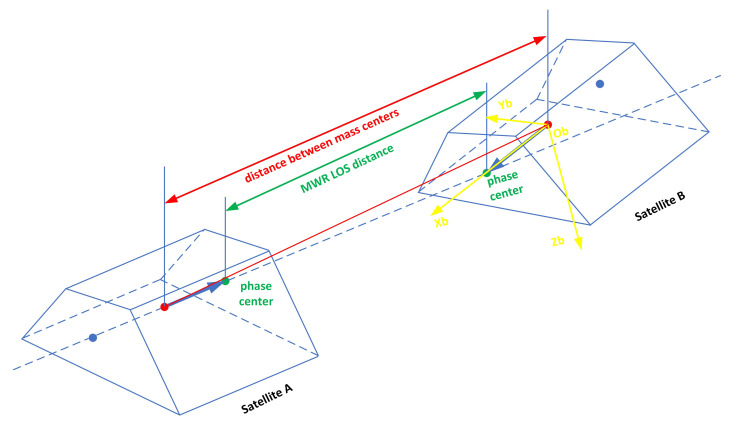
The geometry of LOS distance between leader-follower satellite antenna centers and distance between mass centers (real situation).

According to MWR and attitude measurement data, the LOS distance between leader-follower satellite antenna centers is transformed into the distance between mass centers. The distance transformation error from antenna phase center to centroid is related to the stability of antenna phase center, centroid stability, MWR Boresight (the MWR LOS that is the vector from the satellite centroid to the phase center of MWR antenna) installation accuracy within X-axis of satellite coordinate system, the attitude measurement accuracy, and the distance stability between the antenna phase center and the centroid. The principle of distance transformation is shown in Figure 1 and Figure 2.

### 2.2. MWR System Design and Development

#### 2.2.1. MWR System Composition

The MWR is a DOWR microwave phase accumulation ranging system, and adopts mainly three measures to ensure ranging accuracy: (1) Correction of ionospheric effects by dual frequency measurements; (2) Synchronous dual one-way ranging technique used to cancellation the medium- and long-term stability of frequency source; (3) Improving phase measurement accuracy by differential frequency phase measurement method.

The composition block diagram of one on-board MWR system equipment can be found in Figure 2 of [26], not shown here. MWR system is composed of the same ranging equipment on two leader-follower formation satellites, except for the difference of ultra-stable oscillator (USO) frequency of 66Hz. The main components of MWR equipment for each satellite of MWR system are as follows:

1. Single horn antenna, used to transmit and receive K/Ka band dual frequency microwave signals (shared by both transmit and receive dual frequencies);
2. USO is used as the frequency reference of the whole system;
3. The K/Ka transmitter is used for up converting the reference frequency to generate K/Ka transmitting carrier frequency;
4. The K/Ka receiver uses the carrier frequency of the K/Ka transmitter as the mixing frequency, down converts the receiving carrier to the designed phase measurement frequency, about 500 KHz;
5. BeiDou receiving antenna and BeiDou receiving channel take USO as frequency reference, completing BeiDou B1C/B2a signal receiving, frequency down conversion, filtering, amplification, and, finally, output intermediate frequency (IF) signal to digital signal processing unit;
6. The digital signal processing unit receives carrier phase measurement with frequency of about 500 KHz from K/Ka receiver, completes high-precision carrier phase extraction, and receives IF signal from BeiDou B1C/B2a receiving channel, completes acquisition of pseudo range and carrier phase observation and navigation calculation, finally outputs scientific data and navigation data to satellite platform on-board data handling (OBDH) system. The distance, velocity, acceleration, and other information between formation satellites can be obtained through ground processing.

#### 2.2.2. MWR System Errors Analysis

In order to achieve microns level ranging accuracy performance, K/Ka band carrier frequency is selected to obtain sufficient carrier phase detection capability and ranging resolution, such as detection accuracy of $10^{-3}$∼$10^{-4}$ cycles for 24 GHz carrier signal, ranging accuracy can be achieved to 12.5∼1.25 μm. The selection of system working frequency should consider the requirements of ranging accuracy, USO frequency, and microwave direct multiple locking, and the requirements of maximizing ionospheric time delay correction.

Moreover, the ranging in K/Ka band high-frequency using direct phase measurement cannot meet the requirements of high stability and high precision, due to the influence of various internal parasitic disturbance. In practical, the differential frequency phase measurement method is used, which mixing the local transmit carrier and receive carrier to achieve high precision and high stability phase measurement at low frequency (500∼700 kHz). In order to realize the consistency of MWR equipment of the two formation satellites, the USO of the reference frequency source is set differed about 66 Hz for the MWR system of the two satellites. Finally, the MWR system of the two satellites can achieve high consistency of equipment, except USO, and the DOWR can be used to realize the cancellation of related common errors of the deployed MWR equipment.

**Figure 3:**
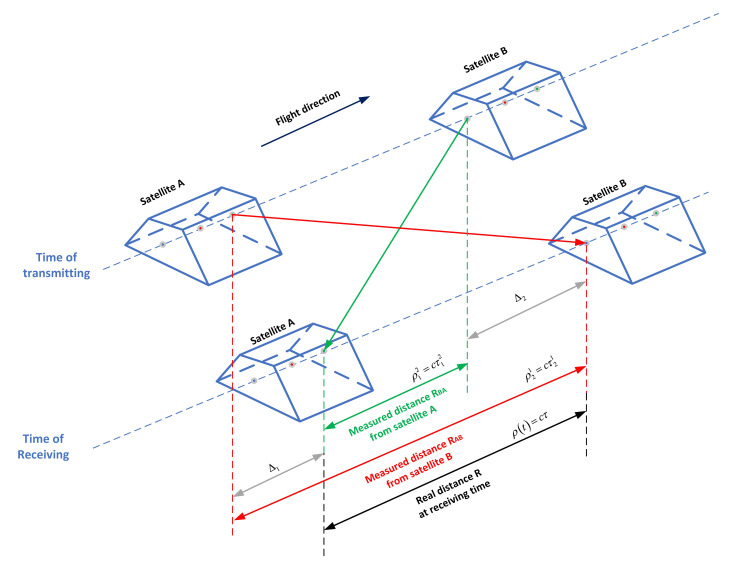
Schematic diagram of light-time correction. The black arrow denotes the real distance ρ(t) at receiving time, while the red and green arrows denote the measured distance $\rho_{2}^{1}$ and $\rho_{1}^{2}$.

The main influencing factors of MWR measurement accuracy are time delay stability of radio frequency (RF) channel, antenna phase center stability, multipath error, ionospheric delay error, USO phase noise, time-tag error, system noise, etc., and two error sources mainly related to the platform design of the satellite [25]:

1. Time delay stability of RF channel and stability of antenna phase center, a crucial segment to ensure the high precision of measurement of the MWR system, which are mainly affected by the temperature stability. Since the MWR system measures inter-satellite distance change and its rate, there is no strict requirement for equipment absolute time delay, but it requires equipment to have high delay stability, including stability of antenna phase center and time delay stability of RF channel. Those two stabilities are highly sensitive to the change of the ambient temperature, therefore it is necessary to make high-precision temperature control method key components of MWR equipment. The satellite platform is required to conduct high-precision temperature control for MWR, providing there are ideal environment conditions for the highly sensitive oscillator and antenna material structure;
2. Multi path error. If the geometric relationship between transmitter and receiver changes, multipath will introduce a variable range offset which is difficult to correct. Here we solve it by using the typical antenna and satellite front panel and setting the satellite pointing requirements through high-precision attitude control.
  The other MWR related error sources including:
3. Ionospheric delay error. The K/Ka dual frequency measurement technique is adopted to correct the influence of ionospheric refraction, aiming to achieve microns level ranging accuracy;
4. Frequency source noise. In order to minimize the influence of the medium and long-term phase noise and inter-satellite relative frequency drift from the USO reference frequency source, the DOWR technique is adopted;
5. Time tag error [31]. To ensure that the change of inter-satellite distance measured at the time tag is equals to the real value in instant, the DOWR technique requires accurate synchronization during ranging process. Suppose we have the nominal frequency of 500∼700 kHz for difference frequency phase measurement method, in order to achieve $10^{-4}$ cycle detect accuracy, the time tag error is required to be less than $10^{-4}/700$ kHz ≈0.15 ns. BeiDou receiver can be used to achieve time synchronization between formation satellites (with synchronization accuracy better than 0.1 ns), so that the carrier phase measurement calibration time of MWR systems can be accurately aligned for two satellites;
6. Instant distance correction error. The distance between two formation satellites is constantly changing during the DOWR process. Assuming that we have the ideal situation of an accurately synchronized MWR system, the receiving time of the two satellites at the end ranging time is accurately synchronized. However, the inter-satellite distance $\rho_{1}^{2}$ obtained by satellite A at the receiving time and the inter-satellite distance $\rho_{2}^{1}$ obtained by satellite B at the same time are different from each other, because the satellite moves at a high speed (about 7.8 km/s for 500 km orbit altitude) during the ranging process (about 1ms for 270 km formation distance). The obtained distances are $\rho_{1}^{2}\neq \rho_{2}^{1}$, and neither of them are the inter-satellite distance at the receiving time, that is, the DOWR is not the same distance when two satellites move at high speed along the same direction, nor the real inter-satellite distance at the receiving time. In this way, the accurate correction of the instant inter-satellite distance is needed when the DOWR measurement is conducted, as shown in Figure 3. Precise orbit determination with the BeiDou receiver can realize precise correction of instant inter-satellite distance, here we call it light-time correction.

Figure 3 illustrated the instantaneous range ρ(t)=cτ at the same nominal time *t*, and the phase-derived ranges $\rho_{2}^{1}=c\tau_{2}^{1}$ and $\rho_{1}^{2}=c\tau_{2}^{1}$ (superscript / subscript 1, 2 denote satellite A, B). With proper modeling of DOWR observable Θ(t), the instantaneous range ρ(t) can be expressed as a sum of phase measurement and TOF correction as [32]:

(1) $$\rho \left(t\right)=\rho_{obs}+\rho_{TOF}$$

with

(2) $$\rho_{obs}=\frac{c\Theta (t)}{f_{1}+f_{2}}$$

(3) $$\rho_{TOF}=\frac{f_{1}}{f_{1}+f_{2}}\dot{\rho }\tau_{2}^{1}-\frac{f_{1}}{f_{1}+f_{2}}\eta_{2}\Delta \tau +\frac{f_{1}-f_{2}}{f_{1}+f_{2}}\eta_{2}\tau_{1}^{2}$$

where *c*: the speed of light; $f_{1},f_{2}$: the transmit signals carrier frequency of satellite A and B (K/Ka); $\dot{\rho }$: the instantaneous range-rate; $\eta_{2}$: the velocity component along the LOS vector; $\Delta \tau =\tau_{1}^{2}-\tau_{2}^{1}$: the time-of-flight difference. On the basis of the magnitude and accuracy estimation to the instantaneous range correction related parameters, the final instantaneous correction accuracy $\delta \rho_{TOF}\approx 5\mu m$ that obtained through [32]:

(4) $$\begin{array}{c} \delta \rho_{TOF}\approx \frac{f_{1}}{f_{1}+f_{2}}\delta \left(\dot{\rho }\right)\tau_{2}^{1}+\frac{f_{1}}{f_{1}+f_{2}}\dot{\rho }\delta \left(\tau_{2}^{1}\right)\\ \;\;\;\;\;\;-\frac{f_{1}}{f_{1}+f_{2}}\left[\delta \left(\eta_{2}\right)\Delta \tau +\eta_{2}\delta \left(\Delta \tau \right)\right]+\frac{f_{1}-f_{2}}{f_{1}+f_{2}}\left[\delta \left(\eta_{2}\right)\tau_{1}^{2}+\eta_{2}\delta \left(\tau_{1}^{2}\right)\right] \end{array}$$

According to the principle of EGFD mission, and the MWR equipment development with system errors analysis in this section, the whole MWR system ranging accuracy can be attributed to two parts: the first one is the LOS distance measurement error between antenna phase centers, using MWR equipment; and the second, distance transformation error from antenna phase centers to satellite centroids. Here we provide those error sources, estimated values, and means of improvement, as shown in Table 1 and Table 2. Finally, with calculation, the system design and error allocation ensure that the ranging error of MWR system can be achieved to less than 50 μm (RMS), within measurement frequency band ($10^{-4}$∼0.1 Hz).

## 3. Precise Time Synchronize and Beidou Receiver

### 3.1. Time Synchronize Solutions of MWR System

The accuracy of MWR system depends on the high precision DOWR measurement. Moreover, the accuracy of DOWR measurement depends on the highly synchronized time-frequency system design [33,34]. With strictly theoretical analysis, it is necessary to use BeiDou or other means to ensure that the synchronization accuracy between formation satellites is less than 0.1 ns, during MWR equipment functioned in-orbit. It is interesting to mention that, GRACE mission carried dual frequency GPS receivers on two satellites to achieve the requirements of inter-satellite time synchronization for K-band ranging (KBR) system [35].

The process of inter-satellite time synchronize accuracy analysis is given as:

Suppose we have the carrier signal observation of satellite A from satellite B at ideal real time spot *t*:

(5) $$\varphi_{1}^{2}=N_{1}+\varphi_{1}\left(t\right)-\left[N_{2}+\varphi_{2}\left(t-\tau_{1}^{2}\right)\right]$$

Currently, the carrier signal observation of satellite B from satellite A at ideal real time spot *t*:

(6) $$\varphi_{2}^{1}=N_{2}+\varphi_{2}\left(t\right)-\left[N_{1}+\varphi_{1}\left(t-\tau_{2}^{1}\right)\right]$$

where φ is carrier measurement, $N_{1}$ and $N_{2}$ are ambiguities, $\tau_{2}^{1}$ is the propagation time of signal from satellite A to satellite B. In the case of ideal synchronization, we have

$$\tau_{1}^{2}=\tau_{2}^{1}=\rho /c$$

For a highly stability oscillator, we have φ(t+δt)=φ(t)+fδt within a short period of time. The results obtained from (5) and (6) were as follows:

$$\varphi_{1}^{2}\left(t\right)+\varphi_{2}^{1}\left(t\right)=2\rho /\lambda$$

Considering clock bias, we have the phase measurement Θ(t) as:

(7) $$\Theta \left(t\right)=2\rho /\lambda +\left(\delta f_{1}\tau_{2}^{1}+\delta f_{2}\tau_{1}^{2}\right)+\left(f_{1}-f_{2}\right)\left(t_{1}-t_{2}\right)+\left(\delta f_{1}-\delta f_{2}\right)\left(t_{1}-t_{2}\right)+\varepsilon_{\Sigma }$$

where 2ρ/λ (circles): the unbiased phase measurement; $t_{1},t_{2}$: time tags of MWR system in satellite A and B; $\left(\delta f_{1}\tau_{2}^{1}+\delta f_{2}\tau_{1}^{2}\right)$: the phase error caused by oscillator noise; $\left(f_{1}-f_{2}\right)\left(t_{1}-t_{2}\right)$: time tag error; $\left(\delta f_{1}-\delta f_{2}\right)\left(t_{1}-t_{2}\right)$: the coupling error term of oscillator noise and time tag; $\varepsilon_{\Sigma }$: other error terms;

With analysis, the dominated measurement error is $\left(f_{1}-f_{2}\right)\left(t_{1}-t_{2}\right)$. By using differential frequency phase measurement method, we have $(f_{1}-f_{2})\neq 0$ (K-band frequency $(f_{1}-f_{2})\approx 500$ kHz, Ka-band frequency $(f_{1}-f_{2})\approx 600$ kHz). Clearly, the measurement error is determined by the time synchronize accuracy, i.e., the value of $\left(t_{1}-t_{2}\right)$. Since MWR require the phase detection accuracy of $10^{-4}$ circle, then we have $\left(f_{1}-f_{2}\right)\left(t_{1}-t_{2}\right)<10^{-4}$, i.e., $\left(t_{1}-t_{2}\right)<10^{-4}/\left(f_{1}-f_{2}\right)=10^{-4}/600\;\mathrm{kHz}\approx 0.16\;\mathrm{ns}$, that is the time synchronize accuracy MWR needed. Several solutions are proposed at early stage:

• First, by using oscillator: The stability of the oscillator is required to reach $2.4\times 10^{-14}/6000$ s, but the performance of the current oscillator available cannot reach this level;
• Second, using homologous rubidium or a cesium clock for precise time-transfer, but the two formation satellites cannot be wired connected in orbit. The precise wireless time-transfer technology with stability and reliability is still under research currently;
• Third, the precise time interval counter is used to determine the precise time differential measurement, still, the two formation satellites cannot be connected in orbit for measurement;
• Finally, the two formation satellites are equipped with dual frequency BeiDou receivers, respectively. Through synchronous sampling, the MWR data are stamped with BeiDou time tag. The down-link transmitted BeiDou observation data are processed after the formation mission on the ground. The relative positioning accuracy of 2–3 cm and the time synchronize accuracy of 0.1 ns can be obtained by using carrier phase differential positioning algorithm, which meets the requirements of the MWR system.

Therefore, the relationship between the MWR system and BeiDou receiver can be summarized as follows: The MWR system achieves the DOWR required time synchronization accuracy of 0.1 ns between formation satellites, by using BeiDou dual frequency receiver. Under the circumstances of 0.1ns time synchronize ensured, the phase measurement error caused by MWR system time tag error will be controlled within $10^{-4}$ cycle.

The engineering realization of MWR inter-satellite time synchronization with BeiDou receiver is as follows: (1) The MWR equipment onboard using homologous a USO frequency source with BeiDou receiver for both satellite A and B, and all frequencies are generated from USO; (2) The MWR system is strictly synchronized with the BeiDou receiver, which is equivalent to the MWR measurement data being stamped with BeiDou time tag; (3) After the BeiDou measurement data of two satellites are transmitted to the ground, the absolute time tag of MWR measurement and satellite position can be determined by using IGS products and precise orbit determination (POD) technology. The positioning accuracy can reach to 2–3 cm and the time determination accuracy can reach to 0.1 ns, which satisfies the time synchronize requirements of MWR system.

### 3.2. Design and Develop of Beidou Dual Frequency Receiver

In order to achieve the accuracy of 0.1ns inter-satellite time synchronization required by MWR system, the BeiDou dual frequency receiver needs to be totally redesigned, with adaptability integrated within the MWR system. In the so-called integrated design, on the one hand, the BeiDou dual frequency receiver needs to share a unified frequency source with the MWR, taking the homologous USO as the frequency reference; on the other hand, it needs to design an integrated hardware platform for the digital processing of the MWR and the receiver, so as to realize the integrated signal processing and eliminate the errors caused by the common channels. The signal processing hardware platform mainly consists of MWR measurement signal processing, BeiDou signal processing, time management unit, and external interfaces [26].

**Figure 4:**
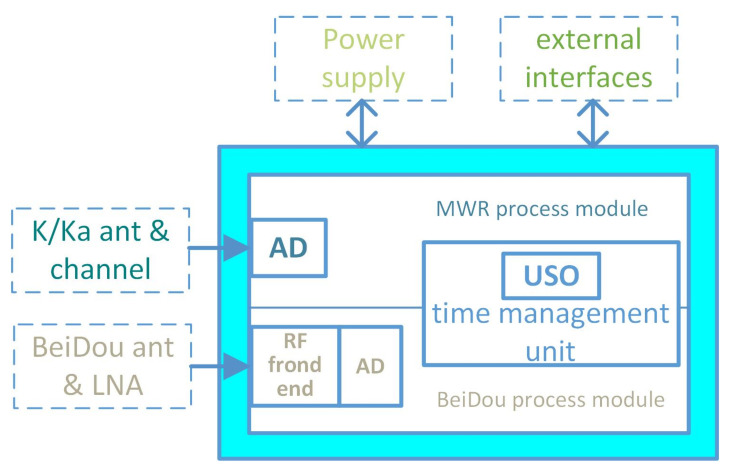
Principle block diagram of integrated signals process hardware platform, including K/Ka band dual frequency MWR process module, BeiDou III B1C/B2a dual frequency navigation process module and external interface module.

The BeiDou dual frequency receiver has been carefully designed and developed that compacted into MWR process module. The receiver prototype photo can be found in Figure 4 of [26], and Figure 4 illustrate the sketchy receiver block diagram.

The design and develop process of BeiDou dual frequency receiver can be summarized as follows:

First, the signal processing scheme in advance: we have to determine whether the signal received by the antenna is filtered, or passing through low noise amplifier first. Before the BeiDou signal is received by the antenna it enters the RF front end, (1) If the first stage is for microwave passive components, such as a filter, then the insertion loss of passive components will be large, which leads to a high noise figure of the whole receiver; (2) If the first stage is low-noise amplifier, the problem of channel blocking should be considered in order to prevent the LNA saturation and the receiver malfunction. The second stage RF filter has the function of frequency and channel selection, which improves the sensitivity of the whole receiver. Finally, the scheme of LNA before filtering is adopted.

Second, gain allocation of system link budget: the signal power received from the antenna is −133∼−90 dBm, and the power level required by the signal processing board is −70∼4 dBm, so, the whole link gain is set to 66 dB. Considering that there may be 40 dB interference signal above the useful signal level, a voltage-controlled attenuator should be added at the front end, possibly 16dB attenuator if necessary. So, the designed system gain link budget is 50∼66 dB.

**Figure 5:**
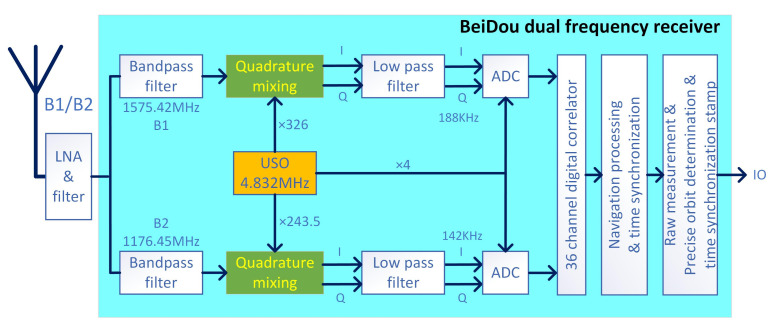
Structure diagram of the dual frequency receiver, including the B1/B2 signals bandpass, quadrature mixing, low pass filter, ADC and the signal process section.

Finally, frequency planning: the 4.832 MHz oscillator signal is properly multiplied for the B1/B2 frequency mixing, as shown in dual frequency receiver structure diagram of Figure 5. Here we choose the zero IF receiver design, i.e., the frequency of local oscillator (LO) is equal to the frequency of RF carrier signal, realizing IF = 0 after mixing, so there is no mirror interference because the mirror frequency is removed. The output of the zero IF receiver is the baseband signal, so there are two orthogonal output signals sending to the baseband for post processing. As shown in green block of Figure 5, two orthogonal LO signals are used to realize the quadrature of the baseband signal after USO frequency multiplication. Compared with the traditional super heterodyne receiver, the zero IF receiver has no intermediate frequency stage, which has the virtues of: directly converting the RF signal to the baseband, and avoiding the image interference problem of the whole system. The zero IF receiver only needs to use the low-pass filter which is easy to be integrated into the system, and greatly reduces the power consumption and cost.

## 4. Test and Simulations

The test of MWR payload is conducted in a precise calibrated six degrees of freedom (6-DOF) moving platform with an accuracy of microns in position and milliarcseconds in attitude orientation, as in Figure 7 of [26]. Two stages of test work have been performed in laboratory: Stage one (Section 4.1), MWR equipment ranging accuracy assessment, with or without integrated BeiDou dual frequency receiver, and DOWR measurement technique test; and stage two (Section 4.2), close loop hardware-in-loop (HIL) simulation that evaluating the relative navigation performance with MWR payload, aiming towards the future of EGFD and DEM spacecraft formation missions.

### 4.1. Assessment of MWR Ranging Accuracy

#### 4.1.1. Test of Time Synchronize Performance

**Figure 6:**
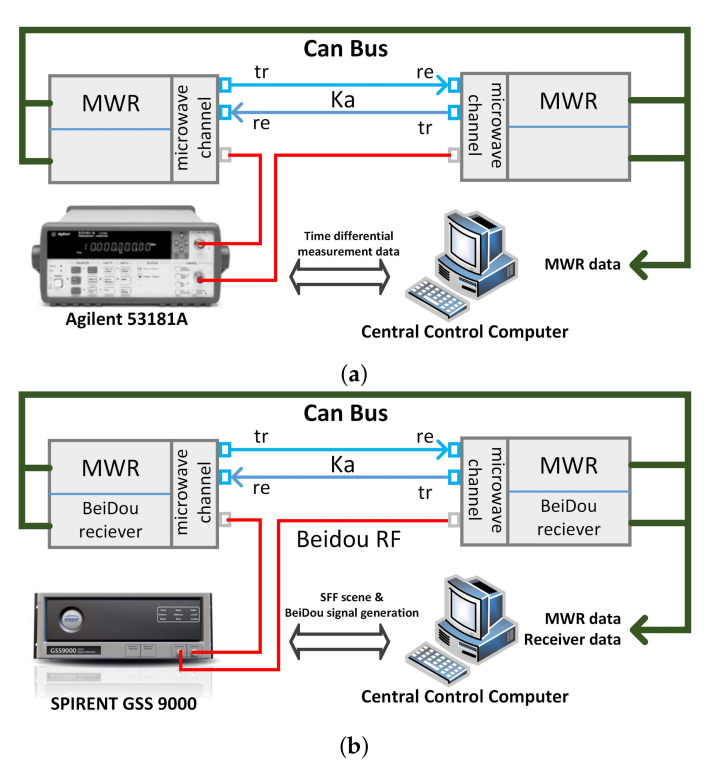
Illustrations of time synchronization test. (**a**) time synchronization test using time interval counter. (**b**) time synchronization test using BeiDou dual frequency receiver.

The performance of time synchronizes provided by BeiDou dual frequency receiver is crucial to the MWR accuracy, therefore, it is worthwhile to conduct experiments of time synchronization prior to the ranging test. Two steps of test work are considered here:

Step one: the time synchronization test using a time interval counter, as shown in Figure 6a. First, the local time difference between the two formation satellites is adjusted to less than 1 ms, next, the 1pps time difference between the two satellites is measured and collected, in real time, by using the precise time interval counter, and transmitted to MWR central control computer through CAN (Controller Area Network) Bus. The central control computer collects MWR phase measurement data of the two satellites at the same time, performing the time synchronization process using the collected time difference measurement data, and obtaining the accurate 0.1 ns time differential result. Finally, resampling the MWR phase measurement data of the two satellites by Lagrange interpolation, based on the value of 0.1 ns time differential result, and the biased distance between the two satellites is obtained by the DOWR process.

Step two: time synchronization test using dual frequency receiver, as shown in Figure 6b. First, BeiDou B1C/B2a dual frequency signal is generated by SPIRENT GSS 9000 simulator. Next, the embedded BeiDou and MWR observation data are collected by central control computer, the BeiDou observation data are used for time synchronization processing to generate time differential data. Lagrange interpolation resampling is performed finally, for MWR phase measurement data of two formation satellites, and DOWR processing is carried out to obtain bias distance between two satellites.

**Figure 7:**
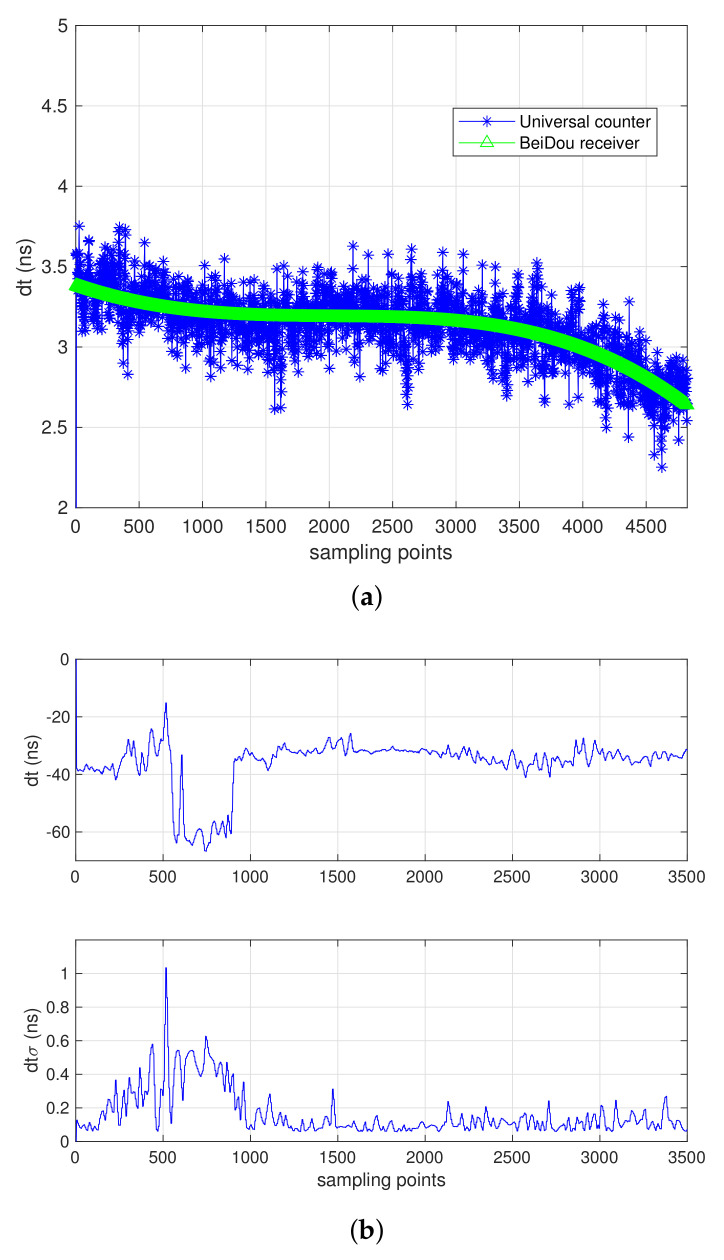
Performance of time synchronization test. (**a**) inter-satellite time synchronization error within 0.1 ns in static test. (**b**) inter-satellite time synchronization error within 0.1 ns in 5 μm/s longitudinal dynamic test.

Here we provide the time synchronize results as in Figure 7, the experiment was performed within one-hour time in 8 April 2021, in static laboratory environment. The blue line and green line in Figure 7a denote the time differential data obtained from time interval counter and BeiDou receiver. The results demonstrated the consistent output of time synchronize performance the receiver can provide, within 0.1 ns after data analysis.

Moreover, a dynamic simulation, using BeiDou receiver only, is also conducted, with a platform moving velocity of 5 μm/s in longitudinal direction. Figure 7b illustrated the time differential results using BeiDou dual frequency receiver. Clearly, 0.1 ns of time synchronization can be achieved after ranging system convergence.

#### 4.1.2. Test of Ranging Accuracy Using DOWR Measurement

**Figure 8:**
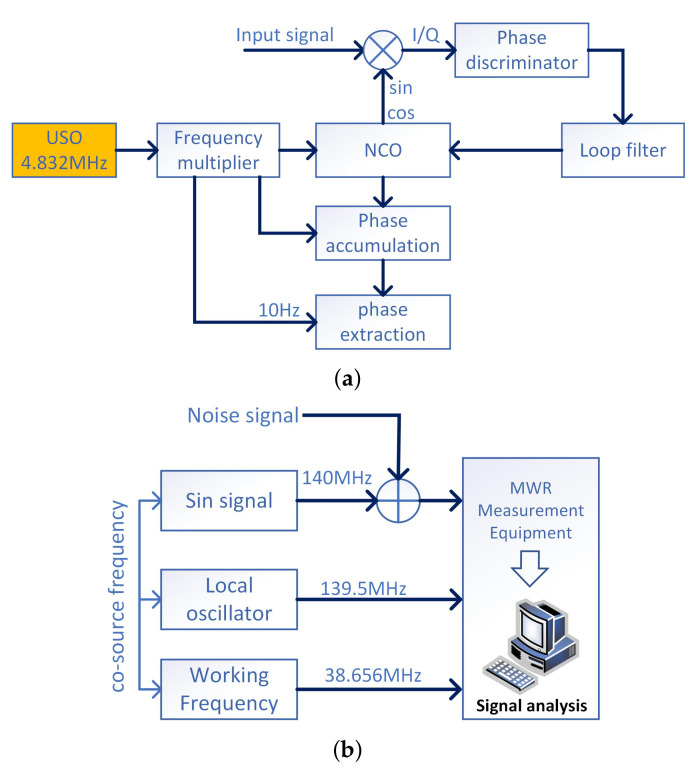
MWR ranging accuracy performance test. (**a**) digital phase locked loop and phase extraction. (**b**) one way ranging test of MWR system.

The test of MWR ranging accuracy is divided into two steps: one-way carrier phase measurement and DOWR measurement. In the MWR system of LEO EGFD mission, the numerically controlled oscillator (NCO) is governed by the local USO. The signal sent to the phase discrimination is used to track and lock the input signal. The error signal generated through phase discrimination, from input signal (about 500 kHz) and the NCO output. After the error signal passes through the loop filter, part of the disturbance caused by thermal noise or other external factors will be inhibited. The signal then enters the NCO again, to adjust its output so that the phase of the output signal approaches to the phase of the input signal until synchronized. Then the phase is extracted from NCO at the rate of 10 Hz and sent to the ground for further processing. The structure of the phase measurement process is shown in Figure 8a.

Suppose we have the two relatively stationary satellites, the frequency of the input signal remains unchanged, then, in the ideal case, after the phase accumulation, the extracted phase measurement should be a constant; when the two satellites are relatively moving, the frequency of the input signal will change, and the extracted phase accumulation value will be varied, so that the distance change between the two satellites can be calculated. Therefore, high-resolution phase estimation technology is essential to achieve micron level measurement accuracy during different SFF moving scenes.

To fully understand the performance of MWR phase measurement through the accuracy accumulation process, here we provide the test results by using a one way ranging structure. The 500 KHz intermediate frequency (IF) phase measurement carrier is generated through the mixing of 140 MHz IF sine wave signal and 139.5 MHz local oscillator inside the MWR hardware platform, as shown in Figure 8b, (to simulate the actual situation of 500 KHz IF phase measurement carrier generated by directly mixing of receiving RF carrier and transmitting RF carrier). After IF signal A/D sampling, it enters FPGA for carrier acquisition, tracking, measurement, and carrier phase extraction, finally outputs to computer for ranging accuracy analysis and statistics.

**Figure 9:**
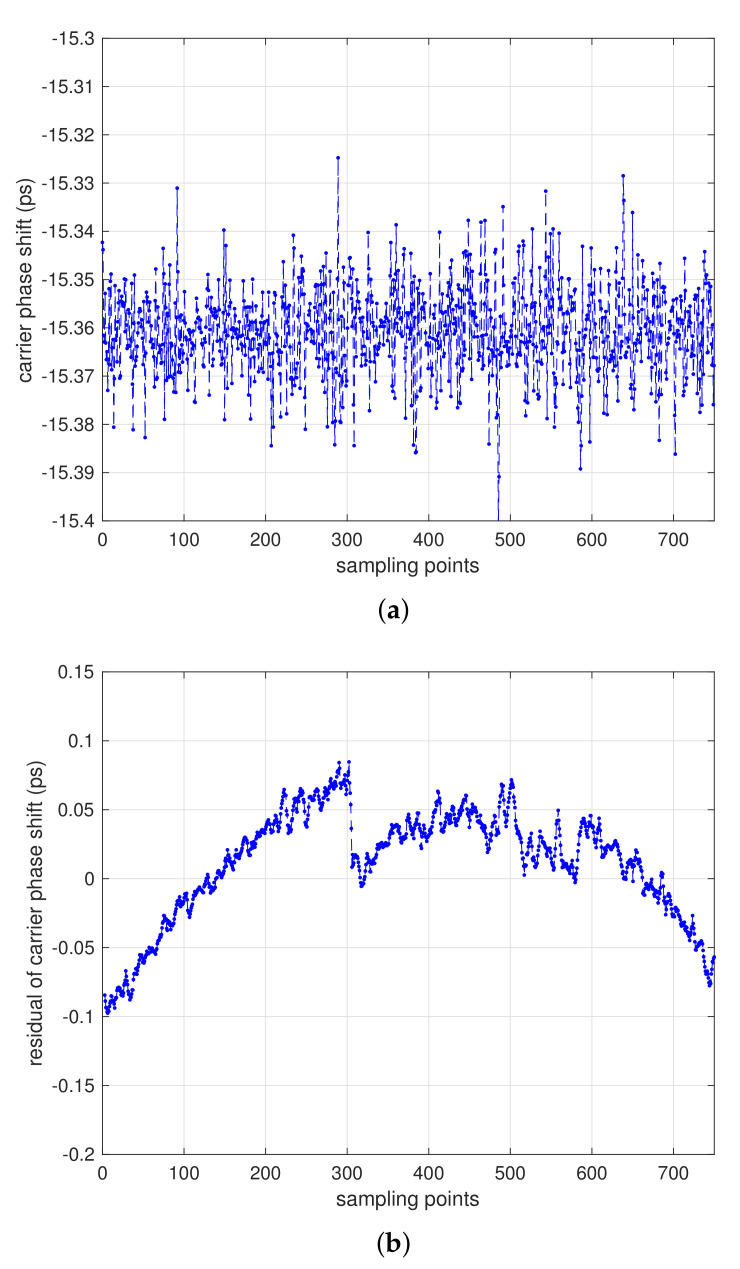
Carrier phase shift performance using one way ranging. (**a**) carrier phase shift results that achieved ranging accuracy of about 6 μm/s (1σ). (**b**) residual obtained by the difference between the measured phase shift data and the curve from first-order fitting.

Suppose we have the constant input signal of 139.999995 MHz, with negative frequency shift of 5 Hz from 140 MHz, Figure 9a illustrated the carrier phase shift measurement results by using one-way ranging experiment. The vertical axis denotes the phase shift (PS) values and the horizontal axis represent sampling points. The average values of carrier phase shift are about −15.33 ps/s, and the ranging accuracy is about 0.021 ps/s under 10 Hz sampling rate, which equals to 6 μm/s (1σ) after data statistic.

Figure 9b shows the residual obtained by the difference between the measured phase shift data and the curve from first-order fitting (the meaning of horizontal–vertical axis is same as in Figure 9a, which reflects the characteristics of clock drift. With strict analysis, the DOWR comparison can eliminate the influence of clock drift on the ranging and velocity measurement.

**Figure 10:**
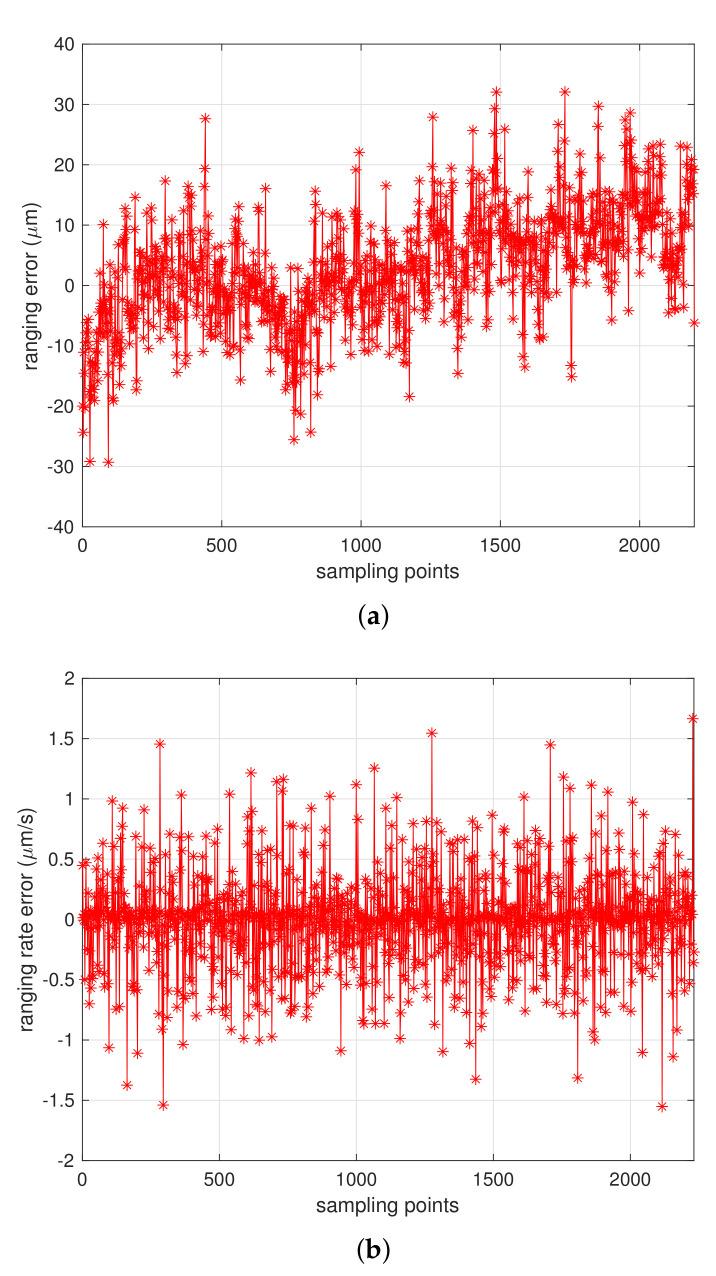
DOWR performance of MWR equipment with data collected from a 6-DOF moving platform under a relative longitudinal velocity of 5 μm/s. (**a**) The ranging error of 2400 samples, achieving less than 40 μm during the test. (**b**) the range rate error that less than 1.6 μm/s during test.

Extensive tests have been conducted to assess the DOWR performance of MWR equipment, under dynamic moving conditions in laboratory. The simulation platform, as introduced before, is precisely calibrated using an optical sensor, that can provide stable moving velocity of 5 μm/s in a longitudinal direction. Figure 10a,b provided the real ranging test results during DOWR ranging experiment in laboratory, as we can see, the ranging error (the differential between real measurement data from MWR and optical moving platform sensor) achieved less than 40 μm during test process, and 1.6 μm/s range rate error was obtained, which verified the microns level ranging accuracy that MWR system provided, demonstrating the ability that could be applied to real space formation missions in future.

### 4.2. Relative Navigation Performance Using Mwr Measurement

The previous ranging accuracy assessment is especially suitable for relatively *stationary* formation missions in space, as EGF detection. Here we entered the stage two test work: close loop HIL simulation that evaluated the relative navigation performance with real MWR ranging payload, aiming to the future spacecraft formation mission.

#### 4.2.1. Hardware in Loop Simulation Platform

The HIL simulation is based on the precise moving platform introduced before. Suppose we have the DEM formation mission with pendulum configuration, constituting a virtual synthetic aperture radar (SAR) in space. The ranging process during the pendulum formation mission is scheduled like this: (1) BeiDou B1C/B2a dual frequency receiver is operated in the whole flight, providing relative carrier phase differential measurement with centimeter level accuracy. It will be regarded as baseline formation distance data in pendulum mission, and considered as initial values when MWR payload turns on. Moreover, BeiDou receiver provide synchronized time-tag of MWR measurements. (2) MWR payload will be operated during SAR Earth observing periods for submillimeter level ranging and precise GNC technology validation. (3) Dual frequency interference optical ranging payload is also used with ranging accuracy of 1 μm (1σ), providing the reference distance data for the validation process.

**Figure 11:**
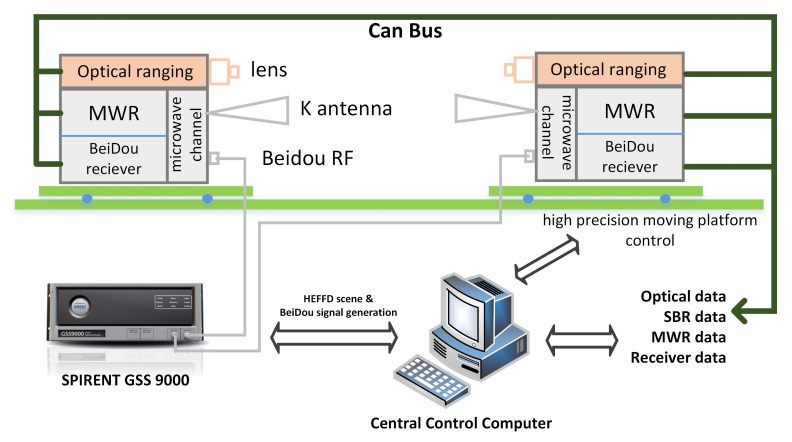
Block diagram of the simulation platform, including a high-precision 6-DOF moving platform, optical/MWR/BeiDou payloads, a SPIRENT GSS9000 simulator, central control computer, and standard CAN bus [26].

The platform is precisely installed with transmitting and receiving payloads, MWR antenna and optical lens equipment, with guaranteed accuracy of fixed position deviation in 2 μm. Figure 11 illustrated the block diagram of whole simulation platform used in this paper. The whole simulation system is coordinated by a central control computer, which conducting the pendulum formation mission scenario management, high precision moving platform control, payload operation, data collection, and analysis. Note a state of art high fidelity SPIRENT GSS9000 simulator is used for the generating BeiDou B1C/B2a signals and real formation mission scene. Dual frequency BeiDou receiver from both formation spacecrafts are connected directly to the simulator, for the purpose of differential carrier phase measurement and precise time synchronization with accuracy of 0.1 ns(1σ).

#### 4.2.2. Mission Orbit Analysis

The formation flying orbit analyzed here, is a virtual pendulum SAR configuration SFF mission that will perform in polar LEO, and the primary concern of such SFF mission is the precise ranging of formation baseline between spacecrafts. The radar based relative orbit motion equations are used here that was suggested by Eggleston and Dunning [36].

Here we provide the baseline circular orbit for SFF mission as: Chief spacecraft orbit altitude: 560 km; inclination: 89.2 deg; argument of perigee: 0 deg; RAAN: 0 deg; true anomaly: 0 deg, and the deputy spacecraft is supposed to be performing follow on pendulum flight relative to chief spacecraft, with distance of about 80 km in-track, and 20 km pendulum amplitude cross-track.

**Figure 12:**
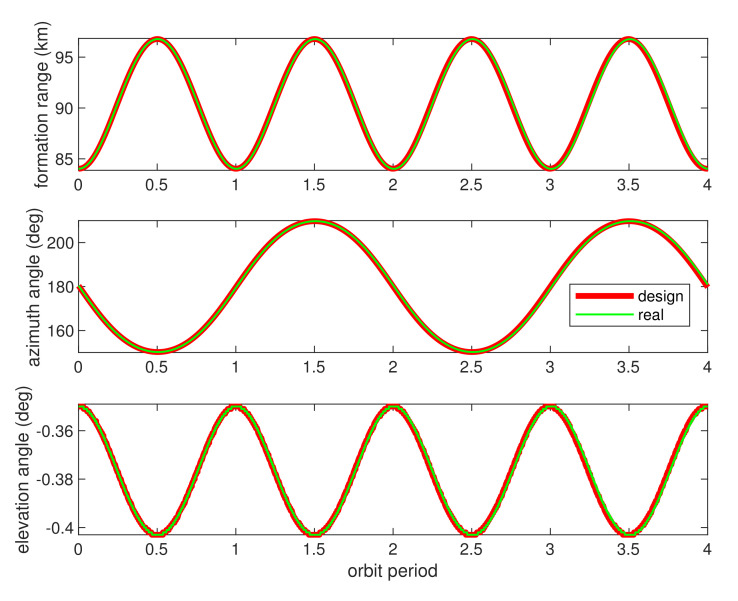
Formation range and azimuth/elevation angles (designed and real values). The red lines denote the designed SFF relative range (**top**), relative azimuth angles (**middle**), and relative elevation angles (**bottom**) during 4 orbit periods, and the green lines denote the same values of SFF in real orbit perturbations.

**Figure 13:**
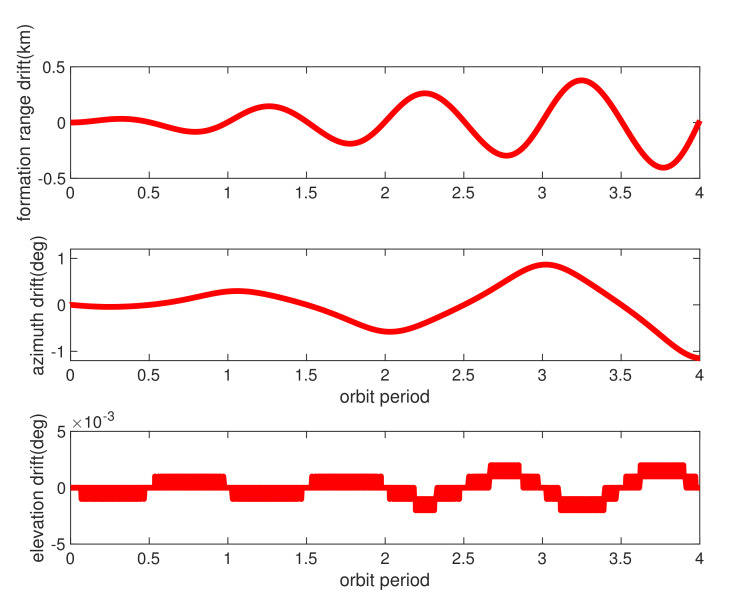
Formation range and azimuth/elevation angles bias under real orbit perturbations. The red lines represent the bias of real formation range drift from the designed values (**top**) and the azimuth/elevation angle drift (**middle**/**bottom**).

For the purpose of formation ranging performance assessment, the chief and deputy spacecrafts are propagated separately in inertial frame, and the relative position and velocity are computed with differences and transformed into the chief relative orbit system, considering as the real orbit values. A precise satellite model is used that similar as GRACE, including structure, surface area, material reflection coefficient, and surface normal vector, etc. [37]. Accelerations of gravitational and non-gravitational are considered, with models shown in Table 3 as:

Figure 12 provided the relative range and azimuth/elevation angle values for 4 orbit periods. Clearly, the orbit calculation starts with relative formation distance of about 85 km, and gradually performing pendulum flight that ranging changed periodically. The bolded red lines in Figure 12 demonstrated the relative orbit values of range, azimuth/elevation angles using dynamic equations of radar model in [36]. Moreover, the green lines in Figure 12 show the same values by using propagated perturbation orbit dynamic using model of Table 3. The results illustrated the real perturbed relative formation orbit drifted quickly from radar model during simulation, and Figure 13 illustrated the drift bias within 4 orbit periods.

According to Figure 12 and Figure 13, the relative orbit drifted dramatically. Formation ranging distance drifted gradually near each far side of pendulum movement, almost reached to −0.5 km at the end of fourth orbit time, as an example, and converged quickly during regression arc. The formation mission configuration can be carefully designed initially, considering detail orbit perturbations. However, sophisticated relative model is not suitable to navigation filter calculation since huge of computation burden on-board for real formation control, as fast autonomous SFF re-configuration GNC system for particular missions. The relative navigation algorithm has to be carefully designed that considering real orbit drift and model uncertainty.

#### 4.2.3. Relative Navigation Performance

The relative navigation filter design in this paper is an adaptive estimate approach that dealing with process noise uncertainty by minimizing a function in real time, which is determined by the difference of residual covariance and residual sequence. Detail introduction of this adaptive estimation algorithm and selection of states variables can be found in [26,38], not shown here.

Some parameters used in this simulation include [26]: Initial date of simulation: April 8, 2021, 14:15:00 (GMT+08:00), sample time interval: 1 s. The spectral densities of the process noise components $w_{x},w_{y},w_{z}$ in relative motion equations, which are each given by $\sqrt{5}\times 10^{-11}$ m/s${}^{3/2}$. The individual standard deviation for initial states η and $\dot{\eta }$ is given by 0.01 km for ϱ(0), 0.1 deg for θ(0), 0.1 deg for ϕ(0), $1\times 10^{-4}$ km/s for $\dot{\varrho }\left(0\right)$, 0.01 deg/s for $\dot{\theta }\left(0\right)$, 0.01 deg/s for $\dot{\phi }\left(0\right)$. 0.05 and 0.05 deg/s${}^{2}$ for leading orbit angular velocity and its rate, and 50 m and 0.01 m/s for the leading orbit radius and its rate.

**Figure 14:**
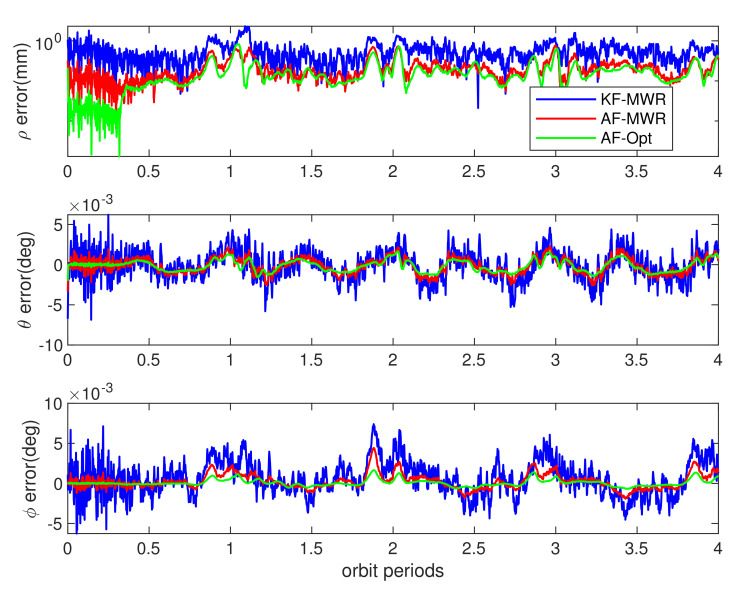
Relative range estimation errors from different payloads and filter algorithms. The blue lines show the relative range and azimuth/elevation estimation errors of MWR payload using the traditional Kalman filter algorithm (KF-MWR); the red lines represent the estimation errors of the MWR payload using the adaptive filter (AF-MWR); and the green lines show the estimation errors of the precise optical payload using the adaptive filter (AF-Opt).

**Figure 15:**
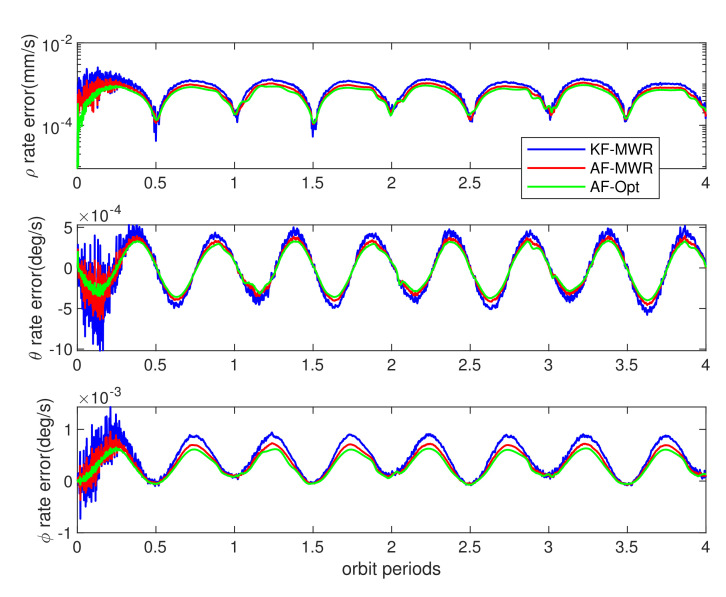
Relative range rate and azimuth/elevation angle rate estimation errors from different payloads and filters (refer to Figure 14 for the denotation of color lines).

The BeiDou dual frequency carrier phase differential measurement was stable during the whole simulation time, by using traditional Kalman filter algorithm. The relative range estimation error (the differential of estimated values and real perturbed values from models of Table 3) achieves a maximum of 1.5 cm. The interesting results are the estimation error using MWR equipment, as shown in Figure 14 and Figure 15, while the blue lines denote the estimation error obtained from MWR, by using traditional Kalman filter. The ranging errors are below 1.3 mm during simulation, as shown in top of Figure 14, and ranging rate errors are within 1.2 μm/s (top of Figure 15). It is worth noting that the relative range rate error changed periodically during simulation: the minimum error occurs during the farthest side of pendulum flight, and divergent gradually to the maximum values around the nearest points, at each orbit period. The reason for this phenomenon can be explained as: the Kalman filter is more sensitive to the changing of ranging rate, in the direction of line of sight (LOS), when the SFF flight around farthest side of pendulum movement, than the nearest side (equilibrium point of pendulum movement), and the same filter trend can also be found in elevation angle rate estimation error in bottom of Figure 15. This is due to the relative ranging errors introduced by orbit perturbation that dominated by the cross-track direction $w_{z}$, and finally influence the range rate and elevation rate estimation results. However, the effect of perturbation $w_{z}$ for azimuth angle vanished, which greatly improved the accuracy of azimuth rate estimation error. The final results are within ± 5$\times 10^{-4}$ deg/s during simulation, as shown in middle of Figure 15.

Clearly, this is not an optimal result for engineering application, especially for the high precision DEM mission. The reason for the filter divergence is this: the formation dynamic model used in the filter, radar model, is not accurate enough for the state’s prediction through recursive calculation at each sampling time. The process noise values of $w_{x},w_{y},w_{z}$ are changing consistently with the orbit perturbations, which need to be adjusted through adaptive approach from measurement update. The red lines in Figure 14 and Figure 15 demonstrate the relative ranging results using adaptive filter introduced in [26]. It is obvious that the estimation accuracy notably improved for both range, range rate, and the relative angles, by using process noise adaptive filter algorithm. The estimation error is less than 0.9 mm for relative range, and the range rate errors are below 1 μm/s during whole simulation time.

The blue and red lines in Figure 14 and Figure 15 illustrated the best filter performance that MWR equipment can be provided with. It is interesting to find what higher filter accuracy can be achieved if a ranging measurement with noise less than MWR is used. As introduced in the previous section, here we also use a dual frequency interference optical ranging payload in this simulation, which offering ranging output with accuracy of 1 μm (1σ). The green lines in Figure 14 and Figure 15 show the estimation errors of same states. The results demonstrated the advanced performance that optical equipment can be provided. However, for real space mission applications, the functional of optical ranging payload rely on highly stable spacecraft platform and accurate pointing mechanism, which limit the scope of application for real-time SFF GNC system. Table 4 provided the error root mean square (RMS) during 4 orbiting periods, for the full states, by using different filter algorithms and ranging equipment. The results clearly demonstrated the effectiveness of adaptive filter that incorporating process noise uncertainty, and submillimeter level ranging accuracy for formation flight in LEO by using MWR technology.

## 5. Conclusions

In this study, we introduced a submillimeter level precise K-band microwave ranging equipment, that is ready for the deployment in spacecraft formation flying missions for Earth’s gravity field detection and digital elevation models in future. Design and development of an integrated BeiDou III B1C/B2a dual frequency receiver is also provided, constituting the 0.1 ns high accuracy time synchronized benchmark for the MWR ranging system. Extensive testing of MWR and BeiDou receivers has been done in a high precision six degrees of freedom moving platform and a hardware in loop simulation system in a laboratory. The MWR system achieved 40 μm and 1.6 μm/s ranging and range rate accuracy during test, suitable for high precision EGF detection formation missions. Estimation of adaptive relative navigation system using pendulum SFF configuration is also conducted, applicable to typical DEM mapping formation mission. The final relative navigation estimation error using MWR are about 0.42 mm (range / RMS) and 0.87 μm/s (range rate/RMS), which demonstrated the promising accuracy for future applications of SFF missions in space.

## Figures and Tables

**Table 1:** The MWR LOS distance measurement error analysis between antenna phase centers.

Error Sources	Error Uncertainty	Means of Improvement
Phase measurement resolution	less than 1 μm	(1) Selection of K/Ka frequency band; (2) Differential frequency phase measurement.
Time delay stability of RF channel/antenna phase center	less than 3 μm/K	(1) Selection control of electronic components/low thermal coefficient machining materials; (2) Minimize the waveguide connection length; (3) Thermo control of MWR equipment.
Multi path error	less than 3 μm	(1) Improve the pointing accuracy of formation satellites and minimize the multipath; (2) Strict requirements for antenna installation.
Ionospheric delay error	less than 2 μm	K/Ka dual frequency measurement and correct
Frequency source noise	less than 3 μm	(1) Low phase noise design of USO and microwave direct multiple locking; (2) Satellite platform control of thermal, magnetic field, radiation and vibration protection; (3) DOWR to eliminate the influence of medium/ long-term stability.
Time tag error	less than 1 μm	(1) Integrated design of MWR and BeiDou, synchronous data sampling with the same time tag; (2) Achieving the precise time synchronization accuracy of 0.1 ns between satellites with post data process; (3) MWR observation data with BeiDou precise time tag synchronous resampling.
Instant distance correction error	less than 5 μm	Corrected according to the precise orbit determination data.

**Table 2:** Error analysis of distance transformation from antenna phase centers to centroids.

Error Sources	Error Uncertainty	Means of Improvement
Attitude pointing control	less than 2 μm	Precise attitude and pointing control support from satellite ADCS and orbit control system.*
Geometric stability	less than 5 μm/orbit	Guaranteed from satellite platform structural design.
Centroid stability	less than 2 μm	Guaranteed from satellite platform centroid stability design.

* ADCS—Attitude Determination and Control System.

**Table 3:** Accelerations of gravitational and non-gravitational models.

Items	Model
GA—the geopotential effect of the Earth	20th order and degree
GA—Sun, and Moon gravities	DE405/LE405 planetary ephemerides model
GA—solid Earth tides	IERS Conventions 1996
GA—ocean tides	Center for Space Research 3.0 model
NGA—the atmospheric drag	NRLMSISE-00 empirical model
NGA—the solar radiation pressure	IERS Standards 1992

Note: GA (gravitational accelerations), NGA (non-gravitational accelerations).

**Table 4:** Statistic of RMS for estimation errors.

Items	Unit	KF-MWR	AF-MWR	AF-Opt
range	μm	935.26	417.91	301.82
range rate	μm/s	1.05	0.87	0.65
azimuth	deg	$4.32\times 10^{-3}$	$2.56\times 10^{-3}$	$1.95\times 10^{-3}$
azimuth rate	deg/s	$3.95\times 10^{-4}$	$3.23\times 10^{-4}$	$3.01\times 10^{-4}$
elevation	deg	$4.01\times 10^{-3}$	$2.94\times 10^{-3}$	$1.97\times 10^{-3}$
elevation rate	deg/s	$8.55\times 10^{-4}$	$6.08\times 10^{-4}$	$4.99\times 10^{-4}$

## Data Availability

Not applicable.

## Abbreviations

The following abbreviations are used in this manuscript:

MWR	Microwave ranging
GRACE	Gravity recovery and climate experiment
EGF	Earth’s gravity field
DEM	Digital elevation models
SFF	Spacecraft formation flying
LEO	Low Earth orbit
HEO	Highly elliptical orbit
DOWR	Dual one-way ranging
HIL	Hardware in loop
6-DOF	Six degrees of freedom
CAN	Controller Area Network
GNC	Guidance, navigation, and control
ISL	Inter-satellite-link
NASA	National Aeronautics and Space Administration
ESA	European Space Agency
EGFD	Earth’s gravity field detection
TT&C	Telemetry, track, and command
LOS	Line of sight
USO	Ultra-stable oscillator
KBR	K-band ranging
IF	Intermediate frequency
OBDH	On-board data handling
RF	Radio frequency
POD	Precise orbit determination
LO	Local oscillator
NCO	Numerically controlled oscillator
SAR	Synthetic aperture radar
In-SAR	Interferometric synthetic-aperture radar
GA	Gravitational accelerations
NGA	Non-gravitational accelerations
RMS	Root mean square
